# Quantification of Gas Flaring from Satellite Imagery: A Comparison of Two Methods for SLSTR and BIROS Imagery

**DOI:** 10.3390/jimaging9080152

**Published:** 2023-07-27

**Authors:** Alexandre Caseiro, Agnieszka Soszyńska

**Affiliations:** 1 Research Institute for Sustainability–Helmholtz Centre Potsdam, 14467 Potsdam, Germany; 2 School of Physics and Astronomy, University of Leicester, Leicester LE1 7RH, UK; agnieszka.soszynska@le.ac.uk; 3 Faculty Geo-Information Science and Earth Observation (ITC), University of Twente, 7500 AE Enschede, The Netherlands

**Keywords:** gas flaring, SLSTR, BIROS

## Abstract

Gas flaring is an environmental problem of local, regional and global concerns. Gas flares emit pollutants and greenhouse gases, yet knowledge about the source strength is limited due to disparate reporting approaches in different geographies, whenever and wherever those are considered. Remote sensing has bridged the gap but uncertainties remain. There are numerous sensors which provide measurements over flaring-active regions in wavelengths that are suitable for the observation of gas flares and the retrieval of flaring activity. However, their use for operational monitoring has been limited. Besides several potential sensors, there are also different approaches to conduct the retrievals. In the current paper, we compare two retrieval approaches over an offshore flaring area during an extended period of time. Our results show that retrieved activities are consistent between methods although discrepancies may originate for individual flares at the highly temporal scale, which are traced back to the variable nature of flaring. The presented results are helpful for the estimation of flaring activity from different sources and will be useful in a future integration of diverse sensors and methodologies into a single monitoring scheme.

## 1. Introduction

During the extraction and refinement of crude oil and condensates, some natural gas is withdrawn from the ground [1]. This natural gas can be used for energy production or injected back into the ground, but most often, it is routinely disposed of by flaring, according to the World Bank (https://www.worldbank.org/en/programs/gasflaringreduction/gas-flaring-explained; accessed on 23 July 2023). At the level of a single flare, up to the totality of the natural gas generated as a side product of crude oil extraction can be flared, according to the World Bank (See: https://www.worldbank.org/en/programs/gasflaringreduction#7; accessed on 23 July 2023). Gas flaring occurs globally, the countries flaring the most gas are Russia, Iraq, and Iran (ibidem).

Flaring of natural gas is a polluting process harmful for the natural environment (e.g., [2,3,4]) as well as human health (e.g., [5,6,7]), with up to 360 premature deaths estimated in the US [8]. Flaring also contributes to global greenhouse gas (GHG) emissions (e.g., [9]). In 2020, gas flaring activities caused CO${}_{2}$ emissions estimated at 377 million tonnes (https://www.worldbank.org/en/programs/gasflaringreduction/global-flaring-data; accessed on 23 July 2023). Further emissions include species relevant for both climate and air quality [10], e.g., nitrogen dioxide [11,12] (estimated global emission of 3.6 Tg yr${}^{-1}$ [13]) volatile organic compounds [14,15,16,17], and particulates [18,19,20,21,22,23].

Besides contributing to climate change with the emission of carbon dioxide, gas flaring also emits black carbon, in the range of 73–210 Gg yr${}^{-1}$ [24,25], a particle active in the radiative transfer of the Earth’s atmosphere [26,27], being particularly relevant in the Arctic through local albedo reductions [28,29,30,31]. Gas flaring also emits methane (estimated global emission of 5.6 Tg yr${}^{-1}$) [13,32]. Methane is originally the prominent component of the gas to be flared and its destruction, the combustion efficiency of the flaring process, may not be as efficient as previously assumed [33,34].

The amounts of flared gas are very high; global estimates of flaring activity are in the order of 129 billion (BE) cubic metres a year [25] or even higher (e.g., [35]). Therefore, several international initiatives, which aim to reduce (and later completely eliminate) routine gas flaring, have come to life. Examples of these initiatives are Global Gas Flaring Reduction (GGFR) partnership, or Zero Routine Flaring by 2030 (e.g., [36]). Other initiatives of interest for gas flaring are, e.g., the global methane pledge and the Paris Agreement [37]. Increasing the monitoring of single flares globally is particularly important because of the skewed distribution of activity among flares, with a few flares being responsible for a large part of the emissions [38,39,40]. However, the actual amount of flared gas and emissions is very difficult to quantify globally in a consistent manner over time and space using ground data due to differences in reporting regulations and conditions among and within countries. Additionally, ground data collection is dependent on local conditions: political stability favours continuous reporting whereas wars and conflicts can lead to losses in accuracy or in information altogether, or metering strategies are ill-defined and poorly enforced [41,42]. At the flaring site level, the amount of flared gas, and therefore the success of reduction initiatives, depends on a number of factors, such as the phase of the oil well life (exploration, development and operation) [43], technical optimizations (e.g., [44,45]), local legislation and policy (e.g., [36,42,46]) or the presence of infrastructure to bring the non-flared gas to markets (e.g., [1]) and can only be measured by an accurate reporting of the amount of flared gas; therefore, it requires a globally applicable method that can provide reliable and reproducible results.

Remote sensing techniques can be useful to consistently monitor gas flaring at the global scale, quantify amounts of flared gas and estimate emissions to verify policy compliance [47]. In the last years, several methods for the detection of flaring have been published. Initially, the visible range of the spectrum was used to detect the presence of a flare [48,49,50,51,52,53], but researchers later took advantage of the strong signal a high-temperature subpixel phenomenon leaves in the short and midwave infrared (SWIR and MWIR, respectively) [54,55,56], making a more selective and automated detection of gas flares possible [38,57,58,59,60,61,62,63,64,65,66,67,68,69,70]. Techniques on the detection of gas flares were recently reviewed by Anejionu [71] and Faruolo et al. [35].

Besides detection, flaring activity can be characterised by analysing satellite imagery in the SWIR and/or MWIR ranges. There are many sensors which can be used for the parametrisation of gas flaring, which goes beyond detection in that it quantifies aspects of the flaring process. Parametrisation is here understood as either the derivation of flare characteristics, such as, e.g., its radiative power or temperature, which can subsequently be used to derive the flaring activity, or the direct computation of the flaring activity from remotely sensed physical quantities. Until now, data from the following sensors have been used to characterise gas flaring: the Sea and Land Surface Temperature Radiometer (SLSTR) [64], the Visible Infrared Imaging Radiometer Suite (VIIRS) (e.g., [38,65,72,73]), the Moderate Resolution Imaging Spectroradiometer (MODIS) (e.g., [57,61,63]), and the Bispectral Infrared Optical System (BIROS) [74].

Methods used to characterise flared gas can be grouped into two approaches. The first and most common uses a statistical model fitted between a satellite product and reference, ground-based, flaring activity data [25,38,61]. The second approach aims to describe the combustion of a flare in a physical model [73,75]. Both these approaches can provide reliable results, although their fundamentals differ strongly.

An example of the first approach was initially proposed by Elvidge et al. [60], who used a combination of VIIRS-retrieved flaring area and temperature, agglomerated at a national level (47 countries and two US states) and related it to the reported flaring activity. The method was later further developed to integrate the flare radiance spreading over several pixels, and refactored for the SLSTR sensor by Caseiro et al. [64].

A method of the second approach was developed by Soszynska [75] for BIROS data. The method considered flame parameters (heating value of the gas, temperature of combustion and its efficiency, proportion of energy radiated and emissivity) and sensing parameters (atmospheric transmission, proportion of energy radiated in the bandwidth of interest and ground sampling area), combining them with the radiance measured by the sensor to derive the fuel mass flow. The weakness of the model came from many assumptions made to provide values for the parameters. The parameters of the model were also strongly variable and therefore could differ between geographic locations.

Flaring activity based on remote sensing estimates is uncertain [76,77,78], as are emissions estimates, due to uncertain emission factors [79,80,81,82,83], on the one hand, and to limitations from both the ground-based data used for the fitting (e.g., [32,77,84]), in the case of the calibration-based approach, and the sensors (e.g., [85]), in the cases of the calibration-based and the physico-chemical-based approaches, on the other hand. The former include geographic and temporal biases: the reporting processes, the gas characteristics and the combustion conditions, for example, may have a strong local dimension and limited applicability, become outdated, be inconsistent or highly uncertain. The latter comprises sensor characteristics relevant when observing subpixel phenomena, such as the ground sampling distance, the point-spread function, the spatial and spectral resolutions, the integration time or the dynamic range. For example, although VIIRS data have been used to derive parameters of gas flaring by the World Bank, researchers assessing the dataset and the algorithm stated that small flares are often omitted, temporal sampling is not sufficient for some cases, and due to large viewing angles, distortions may lead to inaccuracies in deriving flaring parameters (e.g., [85]). The uncertainty in the global flaring activity estimates is reflected in the few trend analyses published to this date. Liu et al. [40] found a decreasing trend in offshore flaring activity, yet not suitable to reach the 2030 zero flaring target, whereas Lu et al. [86] found an increasing trend at the global scale. Zhang et al. [43] found a decreasing post-2008 trend in the Gulf of Mexico linked to an increased gas utilization. Brandt [76] found no significant trend between 2012 and 2018 in Brazil, Canada, Denmark, Mexico, Netherlands, Nigeria, Norway, USA and the UK. In their review, Faruolo et al. [35] also found steady flared volumes at the global (onshore and offshore) scale.

Since there is no sensor (or constellation of sensors) able to provide high revisit times with high spatial resolution, as of 2023, using data from different sensors becomes a necessity. Given the urgent need for accurate reporting and reduced uncertainties, as well as the variety of sensors and methods available, in the present work, we analyse the possibility of data fusion of two differing approaches. To achieve this, we investigate the agreement of the methodologies provided by [64] (calibration-based) and [75] (physico-chemical-based) over an offshore region and a period of time of approximately one year. By analysing offshore flares, we limit the influence of variables related to land cover and therefore minimize the uncertainty originating in the background radiation.

A more detailed description of both methods can be found in Section 2, together with a description of the study area and the satellite products used. Section 3 details the results: an exploratory analysis of the SLSTR-based and the BIROS-based methods (in Section 3.1 and Section 3.2, respectively) is followed by the comparison. The comparison is first conducted for the distributions over the region of interest and the whole study period of the retrieved signals (Section 3.3.1), then the analysis focuses on totals at individual flaring locations (Section 3.3.2) and ends with detections coincident in time and space (Section 3.3.3). The results are discussed in Section 4. Section 5 presents our conclusions.

## 2. Materials and Methods

### 2.1. Satellite Products

The flaring activity and characterisation was conducted over the region of interest (ROI) using Level-1b data from the SLSTR instrument on-board the Copernicus Sentinel-3B satellite, and radiance images from the BIROS satellite of the German Aerospace Center.

In our study, we compared two approaches for a common study area and period of time. We used Sentinel-3 SLSTR data for the calibration-based method and BIROS data for the physico-chemical-based method. The study period comprised approximately 9 months between November 2018 and September 2019.

### 2.2. Study Area

**Figure 1:**
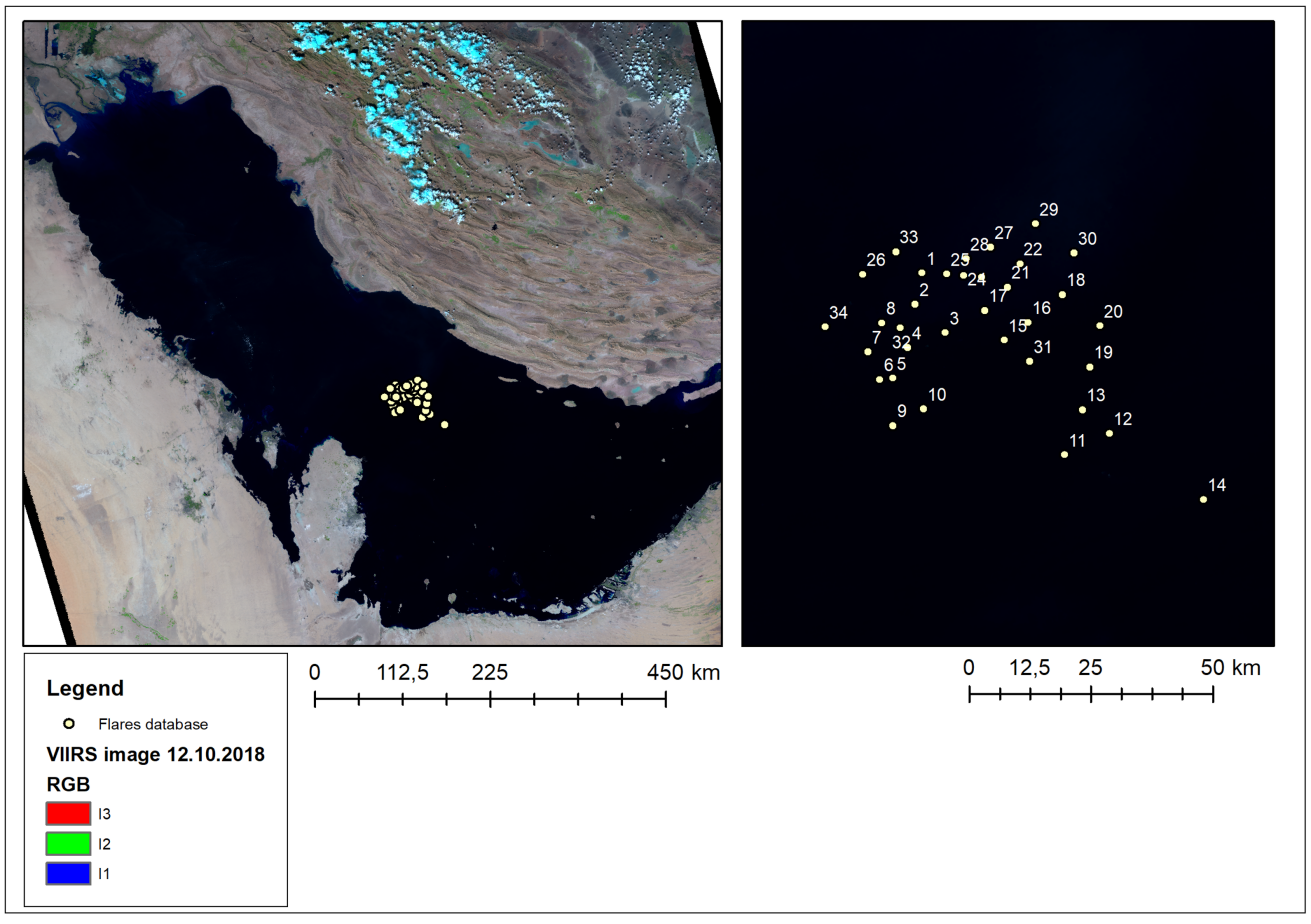
The region of interest, with the location of the gas flares in the geodatabase [75].

The ROI was an offshore area of the Persian Gulf between Qatar and Iran. In this region, a geodatabase of gas flares was created, based on the photointerpretation of high-resolution imagery from the LANDSAT-8 panchromatic band (15 m GSD). The database contained 34 gas flares with their exact geolocation. The flaring locations are shown in Figure 1.

The selected ROI allowed the study of a relatively large number of gas flares within a small area (thus minimizing the variation in the gas composition) under cloud-free detections and against the sea as background, which is more homogeneous in terms of surface temperature than other environments. The latter reason minimized the influence of surface phenomena other than flaring and maximized the influence of the factors related to the sensors and the methodologies in the comparison. As such, the focus was on the differences derived from the instrumentation and the methodologies.

The location of offshore gas flares was known a priori when applying the physico-chemical-based method to BIROS scenes. For the calibration-based method, a detection step was performed on SLSTR scenes (revisit frequency of once per night) prior to conducting the activity determination. The detection was based on an elevated signal in the 1.6 μm band at nighttime, given cloud-free conditions in that pixel at its surroundings. See Elvidge et al. [38] and Caseiro et al. [64] for more detail on the gas flare detection.

### 2.3. Calibration-Based Method

The calibration-based method was an adaption of a method developed by Elvidge et al. [38], who used a statistical model to determine flaring activity. The model assumed that despite differences in efficiency of gas combustion and variations in gas heating value, there should be a reasonably consistent relationship between reported flared gas volumes and estimated radiative power.

The independent variable of the linear function introduced by Elvidge et al. [38] was given by a modified Stefan–Boltzmann equation. An exponent was applied to the flame area term in order to account for the nonlinear relationship between the power radiated and the flared gas volume in large flares. The flame temperature was obtained by adjusting the sum of two Planck curves (one for the background and one for the hot source) to nighttime radiances observed by VIIRS (visible, SWIR and MWIR bands, 6 bands in total) in the VIIRS NightFire (VNF) algorithm. The flame area was also an output of the dual Planck curve fitting and was subsequently adjusted for possible side-viewing effects before applying the modified Stefan–Boltzmann equation. The dependent variable of the model was the volume of flared gas. Reference data used for fitting the model consisted of 47 reported upstream flares (plus venting, assumed as negligible) at a country-level and state-level reporting for two US states (Texas and North Dakota). The limitations of the approach have recently been reviewed by Schade [77].

Caseiro et al. [64] adapted the methodology to the SLSTR sensor, with modifications to the detection of gas flares and the retrieval of the flame’s temperature and area. The main modifications pertinent to the determination of gas flaring activity was a clustering of adjacent hot pixels and the use of thermal infrared (TIR) bands, besides the SWIR and MWIR bands, for conducting the dual Planck curve fitting. The latter was considered in version 4 of VNF [67]. A year-long comparison between the original [38] and the adapted [64] versions at the global scale can be found in Caseiro et al. [25].

The total number of Level-1b products analysed was 238, with sensing dates between 21 November 2018 and 6 September 2019. A set of 30,540 hot spots were derived in the SLSTR data processing, using the method described in Caseiro et al. [64] and Caseiro et al. [25] (cloud-free hot pixel and more than 3 cloud-free pixels in its 8 contiguous background pixels), of which 842 were located within a buffer of ±0.025 degrees in latitude and longitude from an offshore flaring location listed in the geodatabase (see Section 2.2).

### 2.4. Physico-Chemical-Based Method

In the physico-chemical-based method, the total instantaneous energy radiated during the combustion was assumed to be given by the total energy contained in the mass of fuel burned scaled by the combustion efficiency (χ(T)) and the proportion of energy radiated (ρ(T)) (Equation (1)).

(1) $$E_{combustion}=\dot{m}\times LHV\times \chi \left(T\right)\times \rho \left(T\right)$$

Before reaching a spaceborne sensor, the radiation is attenuated by the atmosphere. The attenuation is a function of the wavelength: τ(λ). Sensors such as the SLSTR, VIIRS or BIROS register the radiation in specific bandwidths and only capture the amount of energy emitted within those given spectral ranges. The proportion of the energy radiated within a spectral range is a function of the wavelength, the bandwidth and the temperature: ψ(λ,Δλ,T). Hence, the radiance measured at the sensor in a given band, in units of W sr${}^{-1}$ m${}^{-2}$ μm${}^{-1}$ can then be written as follows:

(2) $$L_{sensor}=\dot{m}\times \frac{LHV\times \chi (T)\times \rho (T)}{4\pi \;sr}\times \tau \left(\lambda \right)\times \frac{\psi (\lambda ,\Delta \lambda ,T)}{A_{pixel}\;\Delta \lambda \;S}$$

The left-hand term of Equation (2) is the measured quantity, whereas the flow mass ($\dot{m}$) is the quantity of interest. The first quotient refers to parameters which depend on the flame, and the second quotient refers to the sensing parameters. Equation (2) can be solved for the mass flow of the flared gas ($\dot{m}$).

The application of this activity quantification approach in the present work made use of the MWIR channel, being therefore not limited to nighttime scenes. Since the method did not include an estimation of the flaring temperature, four distinct flaring temperatures (1200, 1600, 1800 and 2226 K) were assumed. In the present work, too low activity values (below 657 m${}^{3}$ h${}^{-1}$) were not considered.

In the present study, the physico-chemical-based method was applied to scenes acquired by BIROS. BIROS is a sensor installed on a small satellite, within the FireBIRD mission of the German Aerospace Center (DLR). A detailed description of the mission can be found in Fischer et al. [87]. BIROS was developed specifically for fire analysis, so the spectral bands and acquisition modes were adjusted for that purpose.BIROS acquires imagery in two spectral bands: midwave infrared (3.4–4.2 μm) and long-wave infrared (8.6–9.4 μm). Both bands acquire scenes in normal temperature and of hotspots (such as gas flares) using a hot-area mode, which uses a shorter integration time. BIROS is a pushbroom sensor with staggered arrays. Using the staggered arrays, as well as shortening the time gap between the subsequent integration instances, allows the spatial resolution to be 4 times higher than the ground sampling distance related to the physical size of a detector unit. The increased spatial resolution of an image product is therefore 180 m (at the nadir). BIROS has a revisit time of 5 days. The results from the physico-chemical-based method were obtained from 25 cloud-free BIROS scenes sensed from 22 July 2018 to 14 November 2019.

## 3. Results

### 3.1. Detections and Flaring Activity with SLSTR

The processing of SLSTR imagery output the flaring temperature and area, from which the activity was derived (see Section 2.3). The application of the temperature filter described in Caseiro et al. [64] (the derived flaring temperature must be above 500 K and below 5000 K) resulted in 728 flaring detections, each of which associated with the closest of the 34 offshore gas flares from the geodatabase. Eight flaring locations were not detected at all by the SLSTR-based method, while nine were detected eight times or less. Therefore, over half of the flaring locations were detected less than once per month on average.

**Figure 2:**
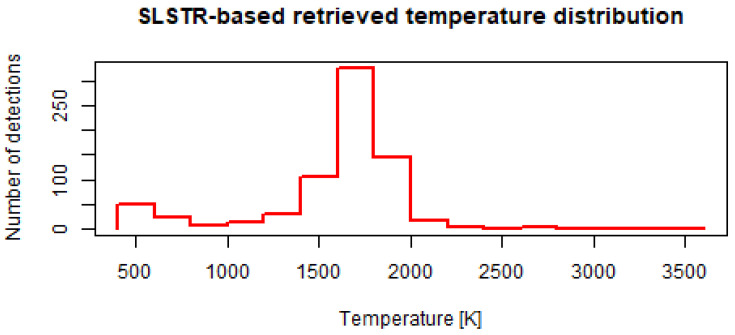
Histogram of the SLSTR-based retrieved flaring temperatures.

Figure 2 shows the histogram of the 728 retrieved flaring temperatures. The distribution shows a mode between 1600 and 1800 K. There is also a smaller mode at much cooler temperatures, around 500 K. Since we only investigated detections at confirmed offshore flaring locations, these low-temperature detections could not be attributed to other hot sources, although it is possible that the very low values were artefacts of the retrieval (e.g., flares with a very small flaring area).

**Figure 3:**
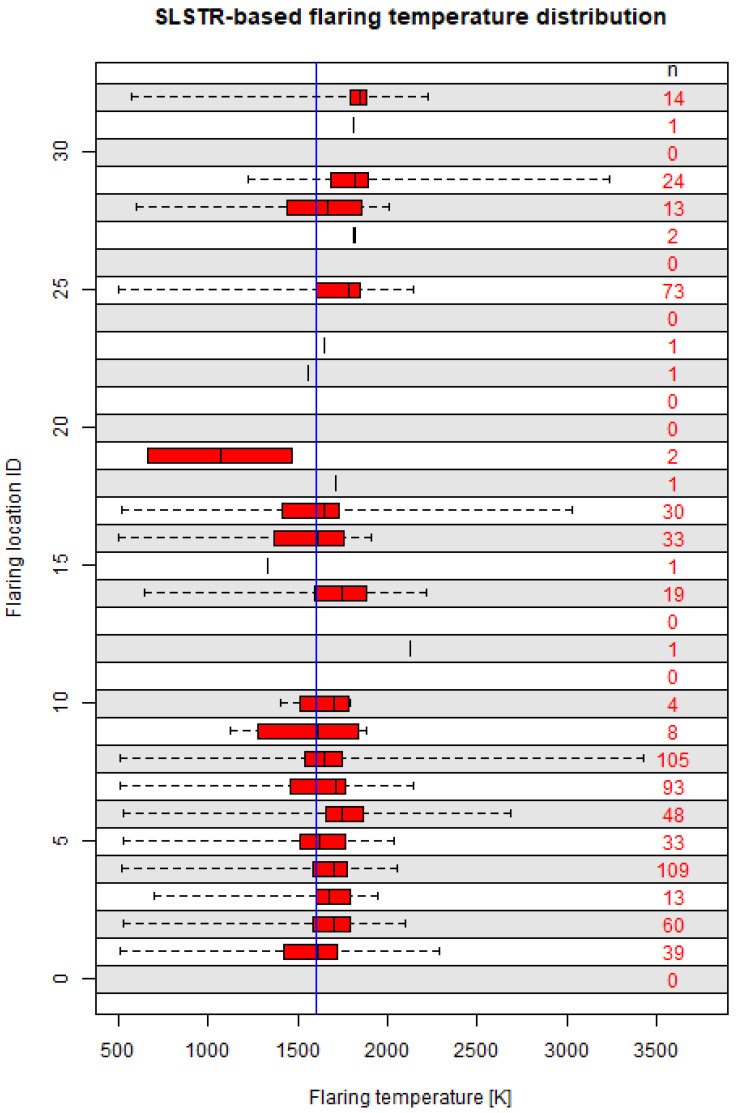
Box-and-whisker (from left to right: minimum, 25th, 50th and 75th percentiles and maximum) plots of the SLSTR-based retrieved flaring temperatures for the individual flaring locations. The vertical line at 1600 K represents the assumed flaring temperature for the BIROS determinations used in this analysis.

Figure 3 shows the temperature distributions at individual flaring locations. Most temperature distributions had their mode in the range 1600–1700 K, with an interquartile range spanning a few hundred K (260 K on average, up to 533 K). Among the 15 flares which were detected more than once a month on average, the variability was substantial, with a standard deviation between 306 and 527 K. The flaring temperature varied with the combustion efficiency, with the maximum efficiency corresponding to the adiabatic flame temperature (2226 K for methane). The number of SLSTR-based retrievals above the maximum burning temperature of methane (2226 K) were only seven, below 1% of the total, showing the physical consistency of the methodology. The variability observed could thus be traced back to a variability in the combustion efficiency, which has been observed in other studies [13,18,34,88]. Several factors affect the combustion efficiency of a flare, such as ambient conditions (atmospheric temperature and pressure, wind), operating conditions (air–fuel ratio in the burner, composition and mass flow of the gas) or possibly the design of the infrastructure (stack diameter) [10,13,21,83,89].

The average mass flow derived from the SLSTR-based detections was 2508 m${}^{3}$ h${}^{-1}$ (3817 kg h${}^{-1}$), with a standard deviation of 3096 m${}^{3}$ h${}^{-1}$ (4713 kg h${}^{-1}$). The variability in the retrieved flaring temperature was propagated at the power of four to the radiative power via the Stefan–Boltzmann equation. The variability was further propagated to the computation of the instantaneous activity: the standard deviation among the 15 flares which were detected more than once a month on average was in the range 341–5071 m${}^{3}$ h${}^{-1}$ (519–7718 kg h${}^{-1}$).

### 3.2. Flaring Activity with BIROS

Valid ($\dot{m}$ over 657 m${}^{3}$ h${}^{-1}$, equalling 1000 kg h${}^{-1}$) BIROS-based determinations were made at 33 offshore flaring locations listed in the geodatabase (see Section 2.2).

**Figure 4:**
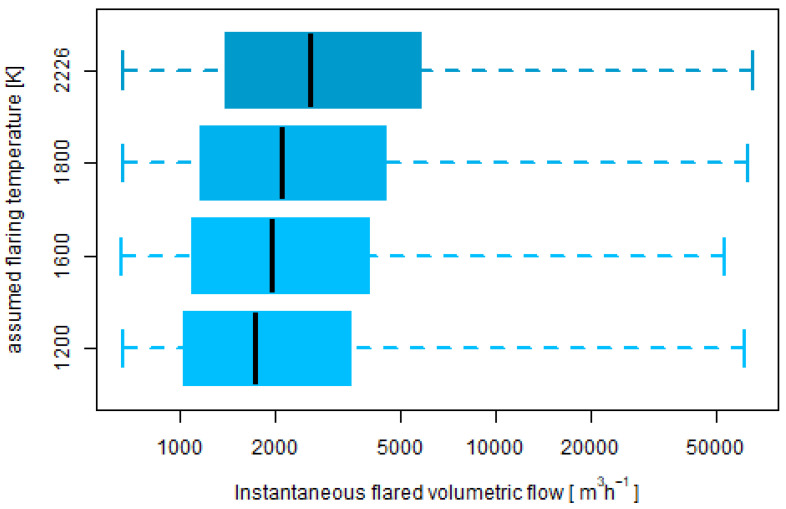
Box-and-whisker plots (from bottom to top: minimum, 25th, 50th and 75th percentiles and maximum) of the determined flaring activity for the four different assumed flaring temperatures.

Figure 4 shows the activity determined from the BIROS-sensed MWIR data for the four assumed flaring temperatures. The average (and median) instantaneous flaring activity were 3826, 4269, 4734 and 5715 m${}^{3}$ h${}^{-1}$ (1734, 1951, 2108, 2603 m${}^{3}$ h${}^{-1}$) for the assumed flaring temperatures of 1200, 1600, 1800 and 2226 K, respectively.

The maximum number of determinations per gas flare was 25 (GF ID 3), with eight locations detected eight times or less (less than once a month on average). Among the 24 flares which were detected more than once a month on average, the variability was substantial: the standard deviation varied between 1534 and 8107 m${}^{3}$ h${}^{-1}$.

### 3.3. Comparison of Flaring Activity

In this subsection, we compare the flaring activity retrieved by both methodologies. First, we look at the regional, long-term activity, then we increment the spatial resolution of the analysis and finally, we further refine it by also increasing the temporal resolution.

#### 3.3.1. Bulk Comparison in Time and Space

**Figure 5:**
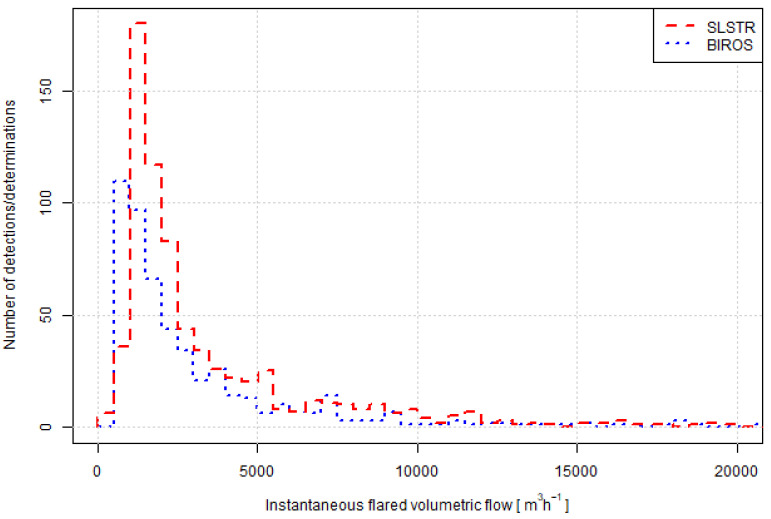
Histogram of the retrieved flaring activities. For the BIROS-based activity values, the assumed flaring temperature was 1600 K. For ease of visualization purposes, activity values were truncated at 20,000 m${}^{3}$ h${}^{-1}$. There were 495 BIROS-based determinations with activity below 20,000 m${}^{3}$ h${}^{-1}$ and 35 with activity above. There were 714 SLSTR-based determinations with activity below 20,000 m${}^{3}$ h${}^{-1}$ and 14 with activity above.

Figure 5 shows a comparison between the histograms of the retrieved flaring activity (728 detections for the SLSTR-based method and 530 determinations for the BIROS-based one). The distributions from both methods compare well in bulk, exhibiting a similar shape, but show different modes: around 750 m${}^{3}$ h${}^{-1}$ for the BIROS-based method, and the following bin around 1250 m${}^{3}$ h${}^{-1}$, for the SLSTR-based method. It is important to note that 750 m${}^{3}$ h${}^{-1}$ is the lowest class in BIROS detections, since values below 657 m${}^{3}$ h${}^{-1}$ are masked as invalid. This discrepancy may evidence either a higher limit of detection (omission of smaller flares) or a positive bias for the SLSTR-based method.

Since we did not have coincidental observations, we investigate in the following subsections how the data derived from both methods at a same location compare (Section 3.3.2), and how data obtained within a short interval of time at a same flaring location compare (Section 3.3.3), in order to investigate the origin of the discrepancy.

#### 3.3.2. Bulk Comparison in Time, Detailed in Space

**Figure 6:**
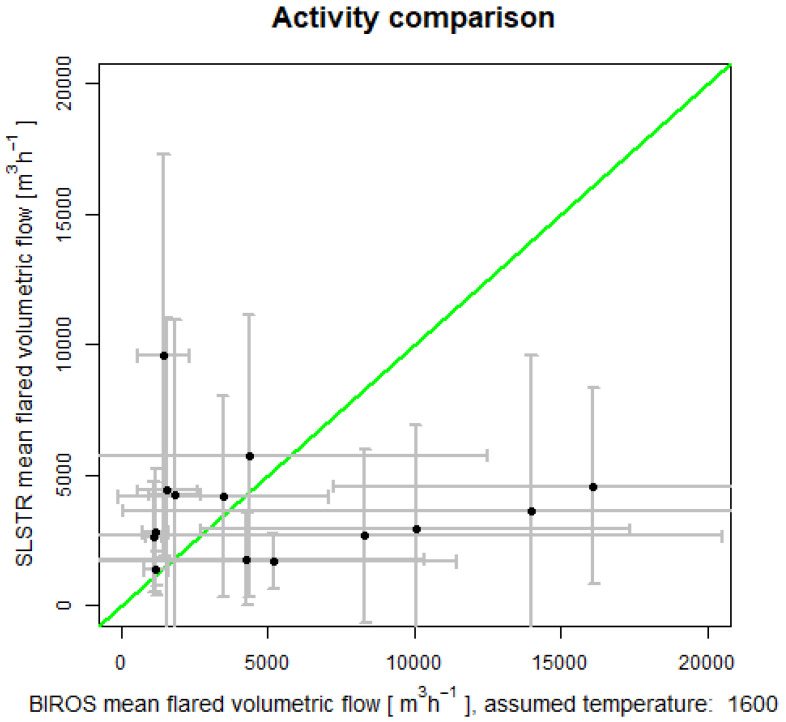
Comparison of the flare-by-flare mean activity (m${}^{3}$ h${}^{-1}$). Only the 14 flaring locations with more than 8 detections for both methods are included. The solid line represents a 1:1 relationship. The error bars represent one standard deviation.

Figure 6 shows the average activity over the study period for individual flaring sites, provided there were more than eight SLSTR-detections and eight BIROS-determinations (approximately one detection per month by each sensor, representing 14 flaring sites in total). Some points show agreement between methodologies and gather around the 1:1 line, but most data points with higher gas flow values are estimated as higher in BIROS than in SLSTR. Another striking feature is the high variability of the data for the same method, as shown by the spread of the error bars which represent one standard deviation, and as reported in Table 1 along with the interquartile range (IQR).

Indeed, the SLSTR-based data (15 flares with more than one detection per month on average, i.e., at least eight in total) showed an IQR of 515–10,216 m${}^{3}$ h${}^{-1}$ and a relative standard deviation in the range 0.4–1.6. The BIROS-based data (25 flares with more than one detection per month in average, i.e., at least eight in total) were less variable in absolute but relatively more variable than the SLSTR-based data: an IQR of 1857–6201 m${}^{3}$ h${}^{-1}$ and a relative standard deviation in the range 0.9–2.1. As stated above (see Section 3.3.1, these numbers also potentially show a higher limit of detection for the SLSTR-based method.

**Figure 7:**
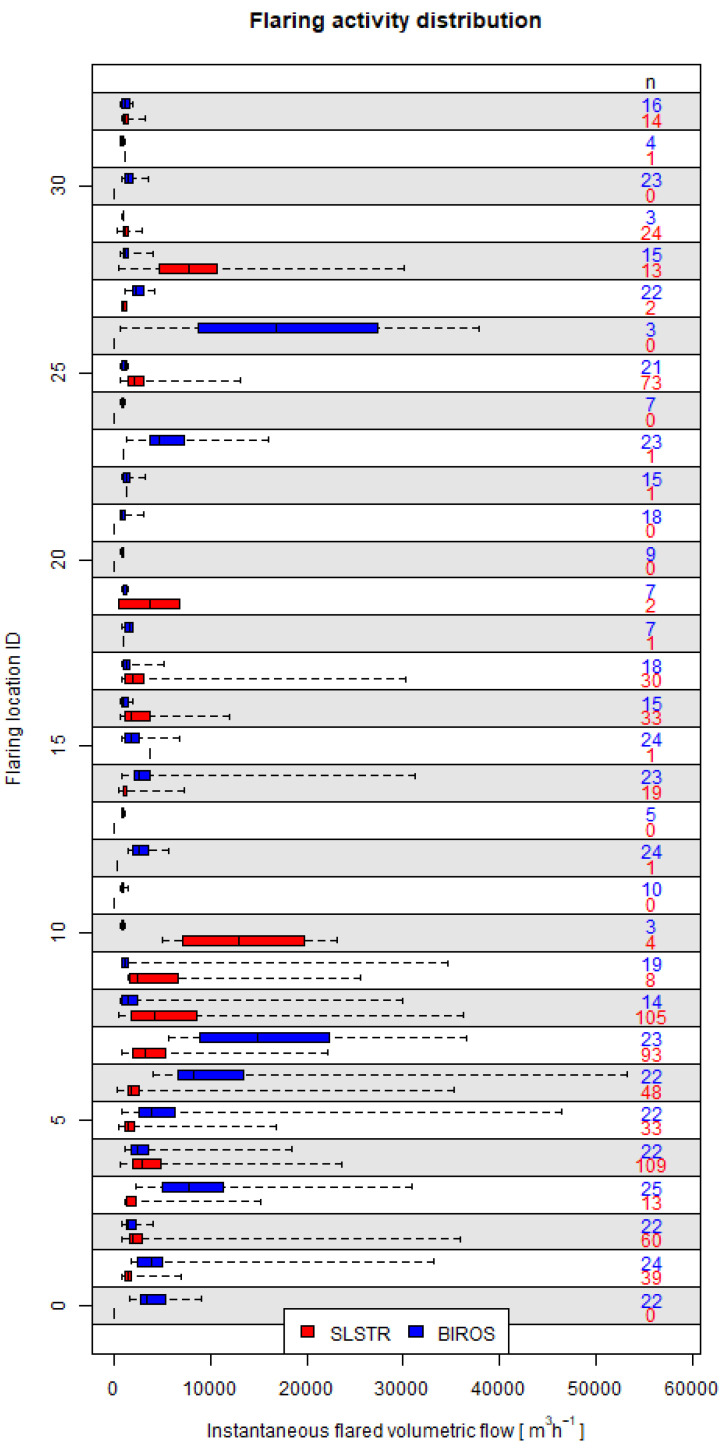
Box-and-whisker (from left to right: minimum, 25th, 50th and 75th percentiles and maximum) plots of the SLSTR-based and BIROS-based flaring activity (m${}^{3}$ h${}^{-1}$) at all the flaring locations.

**Figure 8:**
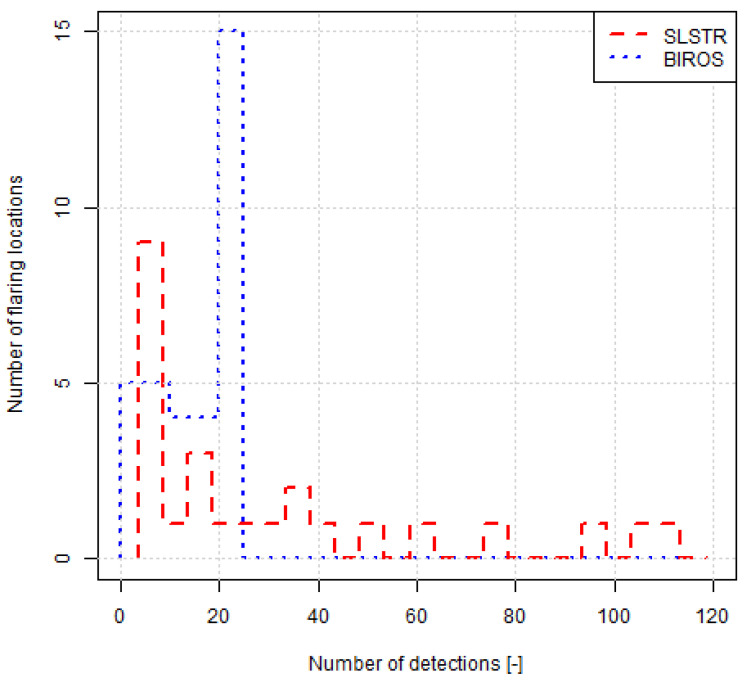
Histogram of the number of samples per flaring location for both methods.

Most points representing the average gas flow calculated with one method lay within the range of the average ± one standard deviation of the gas flow from the other method, the exception being gas flares 7 and 28. For gas flare 7 (Figure 7), there were many more observations from SLSTR data (93) than from BIROS data (23). In the case of gas flare 28, the number of observations was similar (13 and 15 for SLSTR and BIROS, respectively), but the spread of the calculated gas flow was very different in both methods. The distribution of the number of observations by both methods was indeed very different (Figure 8); an increased sampling rate could have lead to minimized differences.

**Figure 9:**
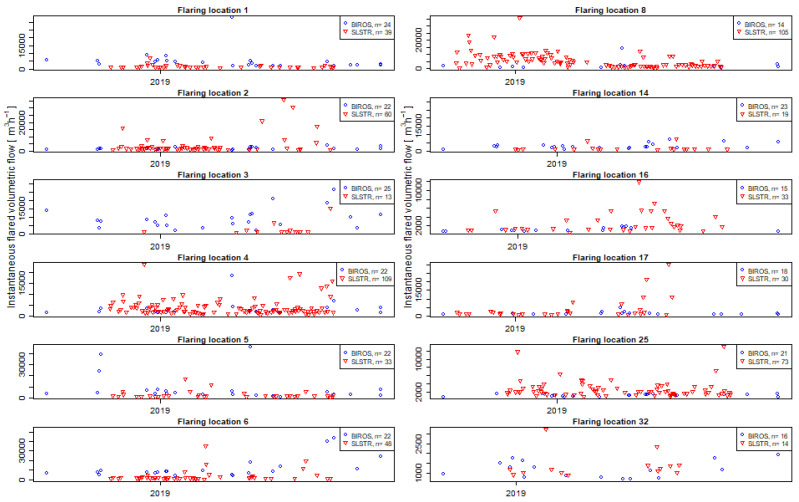
Time series at the 12 flaring locations where the number of observations by both sensors were larger than 8 and whose average instantaneous gas volume flow determined by one methodology was within the average ± one standard deviation of the other. For gas flare 7 (Figure 7), there were many more observations from SLSTR data (93) than from BIROS data (23). In the case of gas flare 28, the number of observations was similar (13 and 15 for SLSTR and BIROS, respectively), but the spread of the calculated gas flow was very different in both methods. The distribution of the number of observations by both methods is indeed very different (Figure 8); an increased sampling rate could have lead to minimized differences.

This shows that a substantial part of the observed discrepancy may originate from the variability within the samples, as can be further observed in Figure 9 (only the 12 flares where the gas flow calculated with one method lay within the range of the average ± one standard deviation of the gas flow from the other method are shown, flares 7 and 28 are not shown) or summarized in Table 1. The observed variability highlights that besides a potential higher limit of detection by the SLSTR-based method, the timing of the sampling is of prime importance when monitoring gas flares.

Table 1 also reports the results (in terms of *p*-values) of the Wilcoxon–Mann–Whitney *U*-test assessing whether the SLSTR-based and the BIROS-based samples originated from the same population (the null hypothesis, H${}_{0}$). H${}_{0}$ (the populations are identical) was rejected (*p*-values < 0.05) for 12 flaring sites and could not be rejected for the remaining 2.

#### 3.3.3. Near-Coincidental Activity

**Figure 10:**
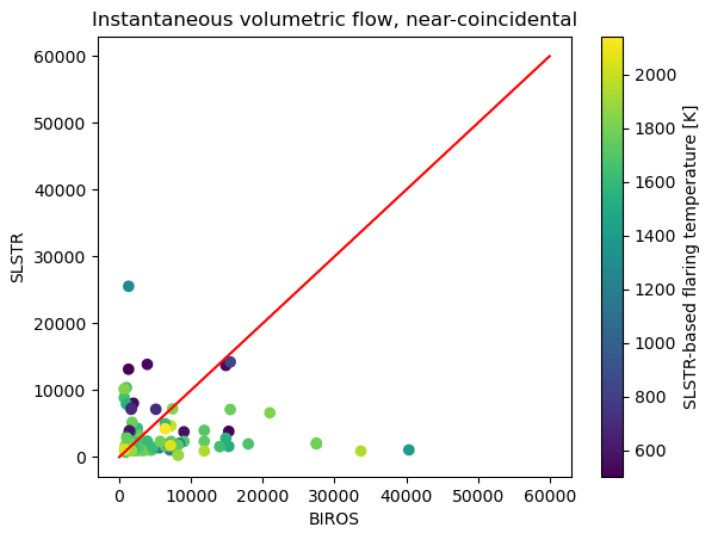
Instantaneous flaring activity (m${}^{3}$ h${}^{-1}$) at single flaring locations for near-coincidental (±1 day) overpasses.

Each BIROS determination was associated with the SLSTR detections that occurred for the same flaring location and within a time span of ±one day. The comparison of the instantaneous mass flow from the resulting 110 pairs of near-coincidental observations are plotted in Figure 10. A set of 34 near-coincidental observations were on the same day, while 76 had an SLSTR detection on the day before or the day after the BIROS determination. For 46 of the near-coincidental observations, the flaring activity between both methodologies was within a factor of two, 17 of which were same-day observations. Figure 10 also shows the SLSTR-based flaring temperature retrieved for each pair. It can be seen that the discrepancy between the methodologies appears to be unrelated to the flare temperature.

## 4. Discussion

Gas flaring is a process harmful to the natural environment, and attempts to reduce this process require a proper method of monitoring. In our research, we compared two methods and two data sources that can be used for this purpose. For the first method, we used data acquired by the SLSTR sensor. The SLSTR-based method has several advantages over other methods published until now, among which the acknowledgement that sensors typically blur the signal of a gas flare into several pixels according to their point-spread function; consequently, we used a cluster-based approach. The SLSTR data can be acquired daily, so the effects of the intermittency of the flares can be minimized.

On the other hand, the physico-chemical method using BIROS data provides a completely different approach: data are not acquired as often, but the higher spatial resolution allows us to better resolve gas flares. The method is based on different assumptions than the SLSTR-based one and the VIIRS-based method published by Elvidge et al. [90]. In the future, a similar comparison between the method published by Elvidge et al. [90] and the physico-chemical method would be interesting, especially since the physico-chemical-based method can be used successfully with VIIRS data, as indicated in Soszynska [75].

In bulk terms, the long-term activity at the level of the ROI (Figure 5) shows some degree of agreement, but the distribution of flaring activity hints towards a possible higher limit of detection for the SLSTR-based method. A significant disparity in the detection limit of one methodology against the other would lead to a decreased number of detections or determinations by a methodology at flaring locations where the activity determined by the other is low. Although some flaring locations exhibit a low number of SLSTR-based detections, a low BIROS-based activity but a high BIROS number of determinations (e.g., flaring locations 0, 22 or 30, Figure 7), other BIROS-based low-activity high-number flaring locations are coupled to higher SLSTR-based detections (e.g., flaring locations 16, 25 and 32). This also occurs the other way around, e.g., flaring location 29 shows a SLSTR-based low-activity high-number flaring locations coupled to low-number BIROS-based determinations, whereas flaring locations 1 and 14 show an SLSTR-based low-activity high-number flaring locations coupled to high-number BIROS-based determinations. The lack of a clear trend is an indication that the detection limit of one method does not differ substantially from the detection limit of the other one.

At the level of individual flaring sites, the long-term agreement (Figure 6) is low. The main question is whether this difference is due to an inherent varying and intermittent nature of gas flaring or the consequence of artefacts for any of the retrievals or sensitivities to the assumed parameters in the physico-chemical model (Equation (2)). The distribution of the points in Figure 6 do not hint towards a possible higher limit of detection for the SLSTR-based method being the main source of the discrepancy, since it would lead to the points being located above the 1:1 line. A systematic high bias for the SLSTR-based method could be the origin of the mode shift in Figure 5, but at the same time, a higher limit of detection for the SLSTR-based method would favour a distribution of the points in Figure 6 above the 1:1 line. Therefore, Figure 6 shows that a possible higher limit of detection or systematic bias by the SLSTR-based method gets diluted in the very high variability of the signal itself. The BIROS-based method could also be subject to a systematic high bias, originating, e.g., in the application of the minimum mass flow rate threshold. However, the distribution of the retrieved mass flow rates (Figure 5), with the mode at lower values for the BIROS-based determinations, do not support this hypothesis. A wrong assumption about the flaring temperature would also lead to a systematic bias for the BIROS-based determinations, but the agreement between the estimated temperature in SLSTR and the assumed temperature in the physico-chemical-based method in Figure 3 does not support that hypothesis.

The discrepancy observed in Figure 6 could stem from a sensitivity to the parameters assumed in the physico-chemical model (Equation (2)), beyond the assumed flaring temperature. The fuel heating value was assumed in our model to be that of methane, but the flared fuel composition, and hence its calorific value, varies in reality [20]. Operational conditions, such as the provision of oxygen or the presence of wind interfere with the combustion efficiency. Atmospheric conditions affect the flame’s emissivity and the atmospheric transmittance. All these parameters are not stated in the calibration-based method but are explicit in the physico-chemical model. The influence of their variations in the discrepancy between methodologies becomes more evident with the discretization of the analysis.

The time series at the fourteen flaring sites with more than eight determinations by both sensors (see Figure 9) show that for some moments in time, the retrieved flaring activity by both methodologies is consistent, while for some others, it is not. This is further indication that the discrepancies in the long-term averages at singular flaring sites originate from variations in the flaring process itself. Although the operational conditions and the legal framing are very different, this finding is in agreement with the intermittence reported by Wu et al. [69] at flaring sites in Texas. An increased sampling frequency, together with a higher timeliness in the overpasses by both sensors, could provide more insight into the question.

As shown above, the long-term activity average for single gas flaring locations is in general consistent between methodologies, within the variability of both methodologies. A further step in the present intercomparison was to analyse pairs of near-coincidental (±1 day) activity retrievals for the single flaring locations. The comparison of the resulting 110 pairs showed that there were large, non-systematic, discrepancies, with only 46 showing a flaring activity within a factor 2. The lack of a systematic nature of the bias further points towards the relevance of sampling and highlights the intermittent and varying nature of gas flaring.

The high variability of the flaring activity at the single flare level found in the present study, together with the skewed distribution of flaring combustion efficiencies and emissions found in other studies [13,18,34,88], calls for an increment in flaring observation frequency by merging data from different sensors and retrievals if the monitoring required for the flaring and GHG emission reduction initiatives is to be implemented.

## 5. Conclusions

In the present paper, we compared two approaches for the retrieval of flaring activity applied to two different sensors, SLSTR and BIROS. The main goal of the comparison was to investigate convergences and discrepancies in order to derive valuable information for future gas flaring monitoring.

The analysis presented here, which is, to the best of our knowledge, the first comparison of flaring activity retrievals in such detail (single detection for a single flaring site) was limited to a particular region with a smooth background in order to focus on the methodological aspects. Further work is needed to characterise how different sensors and methodologies used to monitor gas flares compare throughout different environments. The main weakness of the present study is the lack of coincidental observations from both sensors. We approximated coincidental observations by applying a buffer of one day, but even at that time resolution, the variability, as shown from the analysis from a single sensor, may be of the same order of magnitude or larger than the variability between sensors.

We qualitatively showed that there was no indication of a strong discrepancy in terms of detection limits between both methods. Our analysis showed that the agreement in flaring activity between the methodologies decreased from the regional and long-term scale towards the localized-in-space and near-coincidental-in-time scale. At those scales, the timeliness of the sampling becomes very important. At a near-coincidental (±1 day) time resolution, the variability in the activity retrieved from a single sensor was significant. Thus, even at these offshore sites, the flaring intensity variability and intermittency may be so large as to shadow most of the differences between the two remote sensing activity retrieval methodologies.

Although these findings are a limitation in terms of a methodological comparison, they highlight the necessity to increase the monitoring of flaring activity in terms of time resolution. Although globally, the estimates from different sensors may be convergent and the localized discrepancies identified and characterised [25], the use of flaring activity and emissions in, e.g., atmospheric models which forecast air quality, such as the Copernicus Atmosphere Monitoring Service, require detailed data in both space and time to avoid any sampling bias.

We conclude that further research should focus on integrating the retrievals from different instruments into a single monitoring system. The integration of several sensors in a common effort to detect flaring and quantify its activity as well as to characterise its emissions has the potential to increase detection opportunities and reduce uncertainties. The expansion of the detection to daytime conditions [57,62,91,92] would also be of considerable added value.

## Figures and Tables

**Table 1:** Summary of the flaring activity at the locations where there were more than 8 detections by the SLSTR-based method and more than 8 determinations by the BIROS-based method. For each method, the average volumetric flow (m${}^{3}$ h${}^{-1}$) is given, together with the sample standard deviation ($\hat{\sigma }$), the interquartile range (IQR) and the number of retrievals (n). For each flaring location, a Wilcoxon–Mann–Whitney hypothesis test (*U*-test) was conducted to indicate whether the samples (the BIROS- and SLSTR-derived flared volumes) originated from the same population. The *p*-value of the test is given. The within-variability column indicates whether the average gas flow calculated with one method lies within the range of the average ± one standard deviation of the gas flow from the other method.

	BIROS				SLSTR				*U*	Within
Flare ID	Mean	$\hat{\sigma }$	IQR	n	Mean	$\hat{\sigma }$	IQR	n	Test	Variability
1	5176	6269	2538	24	1691	1067	643	39	1.3 $\times \;10^{-9}$	TRUE
2	1828	895	983	22	4269	6690	1350	60	0.031	TRUE
3	10,035	7310	6418	25	2926	3976	954	13	1.4 $\times \;10^{-5}$	TRUE
4	3463	3587	1699	22	4170	3846	2897	109	0.31	TRUE
5	8309	12,219	3531	22	2655	3325	959	33	4.2 $\times \;10^{-5}$	TRUE
6	13,983	13,912	6032	22	3646	5962	1083	48	8.8 $\times \;10^{-10}$	TRUE
7	16,104	8849	13,375	23	4579	3772	3315	93	2.1 $\times \;10^{-10}$	FALSE
8	4350	8122	1401	14	5743	5402	6712	105	0.0042	TRUE
14	4273	6091	1532	23	1762	1766	347	19	9.7 $\times \;10^{-5}$	TRUE
16	1166	449	622	15	2806	2409	2557	33	0.00086	TRUE
17	1572	1019	641	18	4408	6597	1843	30	0.014	TRUE
25	1103	275	496	21	2631	2093	1613	73	1.1 $\times \;10^{-7}$	TRUE
28	1432	871	564	15	9579	7718	5993	13	4.8 $\times \;10^{-5}$	FALSE
32	1186	426	741	16	1384	650	338	14	0.42	TRUE

## Data Availability

Not applicable.
